# Degrees, Levels, and Profiles of Contextuality

**DOI:** 10.3390/e28050513

**Published:** 2026-05-01

**Authors:** Ehtibar N. Dzhafarov, Víctor H. Cervantes

**Affiliations:** 1 Department of Psychological Sciences, Purdue University, West Lafayette, IN 47907, USA; 2 Department of Psychology, University of Illinois Urbana-Champaign, Champaign, IL 61820, USA; victorhc@illinois.edu

**Keywords:** contextuality, contextuality profile, concatenated systems, degree of contextuality, disturbed systems, level of contextuality, measure of contextuality, nonlocality, undisturbed systems

## Abstract

We introduce a new notion, that of a *contextuality profile* of a system of random variables. Rather than characterizing a system’s contextuality by a single number, its overall *degree of contextuality*, we show how it can be characterized by a curve relating the degree of contextuality (including nonlocality, as a special case) to the *level* at which the system is considered, $\begin{array}{cccccccc} \mathrm{level} & 1 & \cdots & n-1 & n>1 & n+1 & \cdots & N\\ \mathrm{degree} & 0 & \cdots & 0 & d_{n}>0 & d_{n+1}\geq d_{n} & \cdots & d_{N}\geq d_{N-1} \end{array}$, where *N* is the maximum number of variables per system’s context. A system is represented at level *n* if one only considers the joint distributions with k≤n variables, ignoring higher-order joint distributions. We show that the level-wise contextuality analysis can be used in conjunction with any well-constructed measure of contextuality. We present a method of concatenated systems to explore contextuality profiles systematically, and we apply it to the contextuality profiles for three major measures of contextuality proposed in the literature.

## 1. Introduction

There is a consensus that it is not sufficiently informative to merely ascertain whether a system of random variables is contextual. It is also desirable and useful to measure its *degree of contextuality* (for a recent survey of the contextuality literature, see Ref. [1]). We recently proposed that it is also of interest to determine a system’s *first level of contextuality*. The meaning of this term is as follows. Let the joint distribution of any *k* variables in the system be called a *k*-*marginal*. For any *n*, if one characterizes the system by all its *k*-marginals with k≤n, ignoring the higher-level marginals, we say that the system is *represented at level n*. A contextual system is always noncontextual at level 1, and there is a lowest level n>1 at which it becomes contextual. This *n* is the system’s *first level of contextuality*, and at this level, the degree of contextuality, $d_{n}$, can be measured in several known ways. In Ref. [2], this procedure is described in detail for the measure based on the $L_{1}$-distance between a point representing a system at a given level and the corresponding noncontextuality polytope.

We now show that the level-wise analysis can be used in conjunction with any other measure of contextuality (notably, the contextual fraction and the measure involving “negative probabilities”, both extended to apply to systems with disturbance). Moreover, for a system determined to be contextual at level *n*, one can continue to measure its contextuality degree at levels n+1,n+2,…,N (where *N* is the maximum number of variables per system’s context). In this way, one characterizes a system by a *vector of contextuality values*

(1) $$\begin{array}{cccccccc} \mathrm{level} & 1 & \cdots & n-1 & n>1 & n+1 & \cdots & N\\ \mathrm{degree} & 0 & \cdots & 0 & d_{n}>0 & d_{n+1}\geq d_{n} & \cdots & d_{N}\geq d_{N-1} \end{array}$$

that can be called the system’s *contextuality profile*. We discuss ways of exploring the patterns of contextuality systematically. For well-constructed measures of contextuality, $d_{k}$ is nondecreasing in k. This is true for the three measures mentioned above. The tendency of $d_{k}$ to increase with *k* is minimal for the “negative probabilities” measure and maximal for the $L_{1}$ distance measure, with the contextual fraction falling in between.

The structure of the paper is as follows. In Section 2, we remind the reader of the definition of contextuality. Section 3 introduces the notion of a level-*n* representation of a system of random variables. Section 4 presents the main idea of the paper: given a measure of contextuality to define a system’s contextuality profile as the sequence of its contextuality values at different levels. In Section 5, we discuss three specific measures of contextuality that have been proposed in the contextuality literature. In Section 6, we propose a method of concatenated systems for exploring how fast contextuality profiles grow from one level to another. Section 7 presents the results of applying this method to the three measures of contextuality just mentioned. Section 8 contains proofs of the regularities discovered in Section 7. In Section 9, we show that none of the three measures of contextuality is a function of another, by studying contextuality profiles of a special class of systems. Section 10 offers a summary and some questions for future work.

## 2. Definition of Contextuality

Consider a generic example of a system:(2)
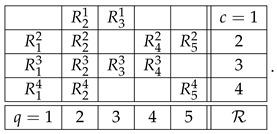

We use our usual notation here. $R_{q}^{c}$ is a random variable recorded in context *c* and answering question *q*. If $R_{q}^{c}$ is defined for a given $\left(q,c\right)$, i.e., if the cell is not empty, we write *q* 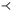 *c* (question *q* is answered in context *c*). The main property of a system of random variables is that all the variables in each row (sharing a context) are *jointly distributed*, whereas no two variables from different rows are jointly distributed (they are *stochastically unrelated*).

A system is *consistently connected* if, for any *q* 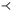 *c*, $c^{\prime }$,

(3) $$R_{q}^{c}\overset{d}{=}R_{q}^{c^{\prime }},$$

that is, any two variables answering the same question are identically distributed. A system is *undisturbed* (or *strongly consistently connected*) if, for any subset of questions $q_{1},\ldots ,q_{k}$ 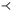 $\;c,c^{\prime }$,

(4) $$\left(R_{q_{1}}^{c},\ldots ,R_{q_{k}}^{c}\right)\overset{d}{=}\left(R_{q_{1}}^{c^{\prime }},\ldots ,R_{q_{k}}^{c^{\prime }}\right).$$

For instance, if the system R above is undisturbed, then

(5) $$\left(R_{1}^{2},R_{2}^{2},R_{4}^{2}\right)\overset{d}{=}\left(R_{1}^{3},R_{2}^{3},R_{4}^{3}\right),\left(R_{2}^{1},R_{3}^{1}\right)\overset{d}{=}\left(R_{2}^{3},R_{3}^{3}\right),\;\mathrm{etc}.$$

The notion of contextuality, extended to include disturbed systems, is described in detail in our previous publications (e.g., Refs. [2,3]). Put briefly, and using the system R in (2), we attempt to construct its *probabilistic coupling*(6)
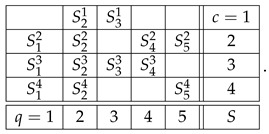

in which all variables are jointly distributed (not just within contexts but overall), subject to the following constraints:

(a) The variables in the rows of *S* are jointly distributed as the variables in the corresponding row of R, e.g.,
  $$\left(S_{2}^{1},S_{3}^{1}\right)\overset{d}{=}\left(R_{2}^{1},R_{3}^{1}\right),\left(S_{1}^{2},S_{2}^{2},S_{4}^{2},S_{5}^{2}\right)\overset{d}{=}\left(R_{1}^{2},R_{2}^{2},R_{4}^{2},R_{5}^{2}\right),\mathrm{etc}.$$
(b) Any two variables answering the same question (e.g., $S_{2}^{1}$ and $S_{2}^{3}$) coincide with the maximal possible probability.

If a coupling of R satisfying (a) and (b) exists, the system R is *noncontextual*, otherwise it is *contextual*.

For consistently connected systems, where the variables answering the same question are identically distributed, the maximal probability of coinciding, e.g., of $\left[S_{2}^{1}=S_{2}^{3}\right]$, equals 1. That is, the requirement (b) then simply makes all variables answering the same question identical. In this case, one can replace the couplings of R with *reduced couplings*,

(7) $$\left(S_{1},S_{2},S_{3},S_{4},S_{5}\right).$$

The system R then is noncontextual if and only if there exists a reduced coupling such that

$$\left(S_{2},S_{3}\right)\overset{d}{=}\left(R_{2}^{1},R_{3}^{1}\right),\left(S_{1},S_{2},S_{4},S_{5}\right)\overset{d}{=}\left(R_{1}^{2},R_{2}^{2},R_{4}^{2},R_{5}^{2}\right),\mathrm{etc}.$$

Let v be a vector of probabilities (all vectors in this paper are columns unless shown as transposed, $v^{⊺}$) that represents the constraints imposed on the distribution of the variables in the system’s coupling. For now, it is not important precisely how it is constructed. Suffice it to stipulate that v uniquely determines the joint distributions of the variables in each context and also ensures that all same-question pairs of variables coincide with maximal probabilities. The existence of the coupling with properties (a) and (b) then means that there is a vector x satisfying the following matrix equation:

(8) $$M\;x=v,\;x\geq 0\;\left(1^{⊺}x=1\right).$$

Here, x≥0 means that every component of x is nonnegative; 1 is a vector of 1s (so that $1^{⊺}x$ is the sum of the components of x). The components of x are probabilities of all possible values of the coupling *S*. Thus, in our example (6), if all variables are dichotomous, there are $2^{13}$ possible values, such as

$$\left[S_{2}^{1}=1,S_{3}^{1}=0,S_{1}^{2}=0,\ldots ,S_{5}^{4}=1\right],$$

and x contains the same number of probabilities. M is a Boolean incidence matrix which tells us which components of x sum to a given probability in v. The reason $1^{⊺}x=1$ is shown in (8) parenthetically is that this condition is satisfied automatically due to the fact that v represents probability distributions.

## 3. Level-Wise Representations

Let context *c* of a system R contain *N* random variables

$$\left(R_{q_{1}}^{c},\ldots ,R_{q_{N}}^{c}\right)=R^{c}.$$

The representation of this context at level n≤N is defined as a system $R^{c}\left[n\right]$ with Nn contexts each containing a distinct *n*-tuple of variables selected from $R^{c}$. For n>N, the level *n* representation $R^{c}\left[n\right]$ consists of a single context containing $R^{c}$. (The reason we denote the row of variables $R^{c}$, in italics, but denote the representing system $R^{c}\left[n\right]$, in script letters, is that we use italics for sets of variables when they are jointly distributed and script letters when they are not, or are not necessarily. We use this notation convention throughout this paper.)

The representation of a system R at level *n* is a system $R\left[n\right]$ consisting of the representation of all contexts of R at level *n*.

As an example, let us consider the second context of system R in (2):(9)
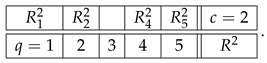

Skipping level 1, which will be discussed later, the representation of this row at level 2 is(10)
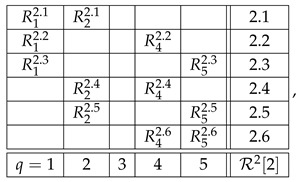

where, for any $q,q^{\prime }$ 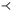  c=2,

(11) $$\left(R_{q}^{2.x},R_{q^{\prime }}^{2.x}\right)\overset{d}{=}\left(R_{q}^{2},R_{q^{\prime }}^{2}\right).$$

In other words, each of the 42=6 rows of the new matrix is obtained by picking from the row $R^{2}$ a subset of two variables, and creating their distributional copy. Clearly, the system $R^{2}\left[2\right]$ representing the row $R^{2}$ at level 2 is an undisturbed system: e.g.,

(12) $$R_{2}^{2.1}\overset{d}{=}R_{2}^{2.4}\overset{d}{=}R_{2}^{2.5}\overset{d}{=}R_{2}^{2},R_{4}^{2.2}\overset{d}{=}R_{4}^{2.4}\overset{d}{=}R_{4}^{2.6}\overset{d}{=}R_{4}^{2},\;\mathrm{etc}.$$

Moreover, the system $R^{2}\left[2\right]$ is noncontextual because it has a coupling coinciding in distribution with $R^{2}$. The system $R^{2}\left[2\right]$ has the same individual and pairwise distributions as the row $R^{2}$, but $R^{2}\left[2\right]$ contains no higher-order distributions (no triples, quadruples, etc.) because joint distributions only exist within but not across the contexts.

The representation $R^{2}\left[3\right]$ of the row $R^{2}$ at level 3 is obtained analogously, by picking from this row 43=4 possible triples of variables and creating their distributional copies:(13)
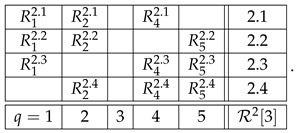

This system, too, is undisturbed by construction, and it is noncontextual as it has a coupling that coincides in distribution with $R^{2}$.

Finally, the level 4 representation of the row $R^{2}$ simply coincides with it because there is only one quadruple we can select from $R^{2}$:(14)
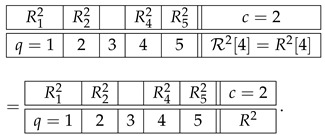

This system is trivially undisturbed and trivially noncontextual.

The higher-level representations $R^{2}\left[5\right]$, $R^{2}\left[6\right]$, etc., of $R^{2}$ also simply coincide with $R^{2}$, which is explained as follows. A representation at level n>1 should always be taken as cumulative, to include not only *n*-tuples but also all lower-level tuples. However, if *n*-tuples exist (the original row contains no less than *n* variables), inclusion or exclusion of the lower-level tuples never influences the contextuality status of the representing system (i.e., whether the system is contextual or noncontextual) or any reasonable measure of the degree of contextuality if it is contextual. So, e.g., the system $R^{2}\left[3\right]$ with an added row c=2.5 that contains the pair $\left(R_{1}^{2.2},R_{2}^{2.2}\right)$,(15)
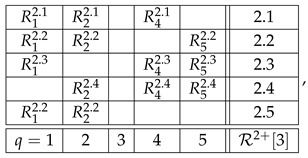

is equivalent to $R^{2}\left[3\right]$ in any considerations of contextuality. This is a general property of undisturbed systems. That is why we defined $R^{2}\left[3\right]$ as containing only triples rather than also pairs and singles, and $R^{2}\left[4\right]$ as containing only quadruples, ignoring triples, pairs, and singles. However, on the next level, 5, no quintuples of variables exist, so we have to include the highest existing tuple, which in this case is the quadruple:(16)
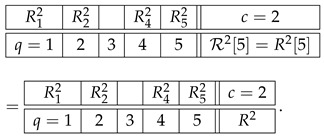

By the same logic, the representation of the first row of the system R,(17)
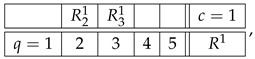

is one and the same at all levels n>1:

(18) $$R^{1}=R^{1}\left[2\right]=R^{1}\left[3\right]=\ldots$$

Let us now explain why we do not consider level 1 representations. In fact, we do include them in the above-given definition of the level-wise representations, but they are always trivially noncontextual, requiring no separate analysis. For our example, the row $R^{2}$, the level 1 representation is(19)
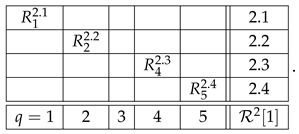

A row with only one variable in it can be removed from any system without affecting its contextuality status.

With the algorithm specified, the level 4 representation of the entire system R is
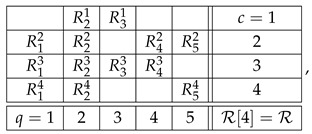

and its level 3 and level 2 representations are, respectively,(20)
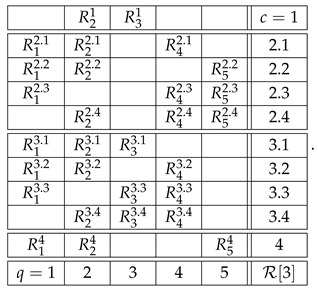

and(21)
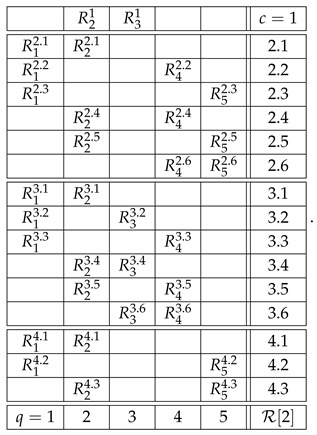


## 4. Profiles of Contextuality

Let us assume that we have a measure of contextuality that applies to any system R (from a sufficiently broad class of systems). Let us denote its value by degR. The main idea of this paper is this: contextuality values of the level-wise representations of R,

(22) $$\deg R\left[1\right]=0,\deg R\left[2\right]=d_{2},\ldots ,\deg R\left[N\right]=d_{N},$$

can be considered the *contextuality profile* of the system R. Here, *N* is the maximal number of variables in a row of R. The values of $d_{N+1}$, $d_{N+2}$, etc., need not be considered because they always equal $d_{N}$. We include the uninformative $\deg R\left[1\right]=0$ as the “anchoring point” of a profile, primarily for aesthetic reasons.

**Figure 1:**
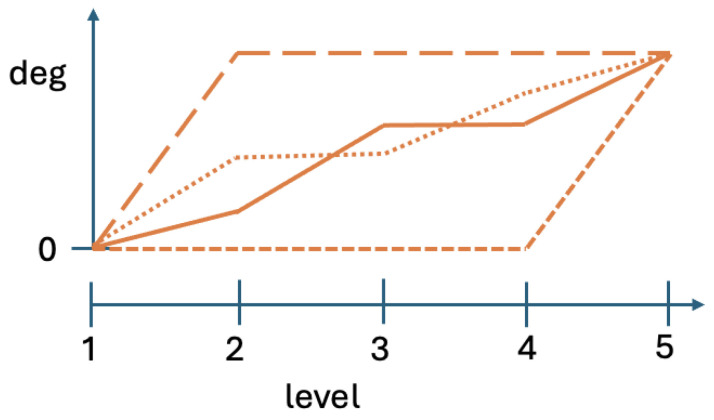
Four possible contextuality profiles with the same final degree of contextuality at level 5.

Figure 1 presents hypothetical contextuality profiles for four systems with N=5. Observe that at level 5 all four profiles have the same value. This common value is the contextuality degree that our measure deg will show for all four systems, because level 5 representations of these systems coincide with the systems themselves. The existing ways of contextuality analysis therefore would treat these four systems as essentially indistinguishable.

In Ref. [2], this idea is partially implemented for the measure of contextuality that we called “hierarchical.” The implementation involves levels of consideration, but the process described there stops at the first contextual level (the smallest *n* with $d_{n}>0$). In essence, for a contextual system, this merely replaces a point measure of contextuality (the single number $d_{N}$) with a two-point one:

(23) $$(n_{\min},d_{n_{\min}}).$$

What we propose now is that

(A) there is no reason to stop at the smallest *n* with $d_{n}>0$, one can compute an entire function $n\mapsto d_{n}$ (n=1,2,…,N), the system’s contextuality profile;
(B) one can do this for any well-constructed measure of contextuality;
(C) computing contextuality profiles for different measures can be an informative way of comparing them.

We propose that a well-constructed contextuality measure should have the following three properties:

1. for any noncontextual system, its final-level degree of contextuality is zero;
2. for any contextual system, its final-level degree of contextuality is positive;
3. its contextuality profile is a nondecreasing function of level.

These requirements are obviously satisfied for the three measures we are going to explore in the next section. However, it is worth mentioning that some seemingly reasonable measures of contextuality may fail them. Thus, in Ref. [3], we describe a measure abbreviated ${\mathrm{CNT}}_{1}$, which, as it turns out, may produce decreasing contextuality profiles. One therefore should consider this measure not well-constructed, and this is the reason we do not include it in the analysis below.

## 5. Three Measures of Contextuality

The three measures of contextuality we are interested in are described in detail in Ref. [3]. Here, we present their brief characterization.

The first measure is the already-mentioned “hierarchical” measure. In this paper we call it the *distance measure*. It is based on the notion of distance between a system and a *noncontextuality polytope*. Consider all possible matrices of a given *format*. The latter is defined by the set of all questions *q*, the set of all contexts *c*, and the relation q 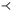 *c*. Two systems of the same format differ in the joint distributions within their corresponding contexts.

Let V be the set of all vectors v such that the equation (8) has a solution:

(24) $$V=\left\{v:M\;x=v\;\mathrm{for}\;\mathrm{some}x\geq 0\right\}.$$

**Figure 2:**
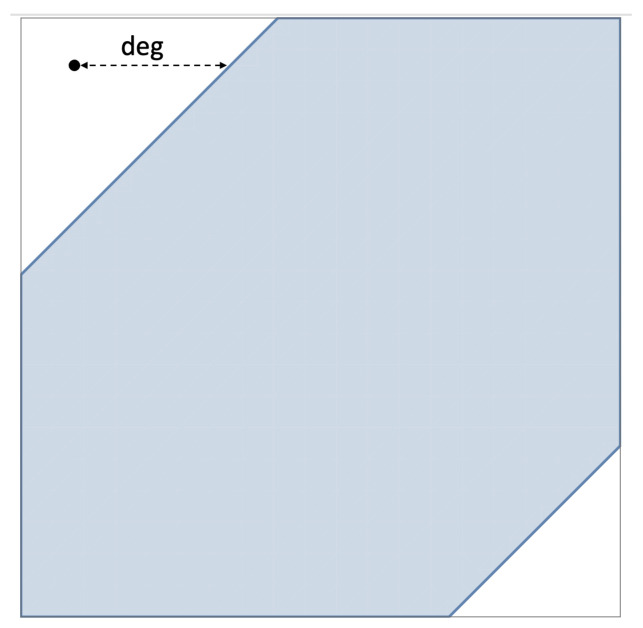
A two-dimensional projection of a vector $v^{*}$ and a noncontextuality polytope V, with the $L_{1}$-distance between them.

It is known that in the space of v-vectors, V forms a polytope, which we call the noncontextuality polytope for systems of a given format (with fixed individual distributions of the variables). If the system we study (let us denote its vectorial representation by $v^{*}$) is contextual, then it falls outside V, and its distance from V can be viewed as a measure of contextuality. When dealing with probability distributions, the distance measure of choice is $L_{1}$, defined by

(25) $$L_{1}\left(a,b\right)=\sum \left|a_{i}-b_{i}\right|,$$

where the summation is over all dimensions of the vector space. Our distance measure is

(26) $$\deg=L_{1}\left(v^{*},V\right),$$

the $L_{1}$-distance between the vector $v^{*}$ and the polytope V, as shown in Figure 2. We denote this measure as ${\mathrm{CNT}}_{2}$, following the nomenclature adopted in several previous publications, e.g., [3].

The next measure of contextuality is based on *quasi-probabilities* (positive and negative numbers that sum to 1). It is obtained by dropping in the definition of noncontextuality (8) the nonnegativity constraint x≥0:

(27) $$M\;x=v\;\left(1^{⊺}x=1\right).$$

This matrix equation is always solvable for x, but some of the components of a solution may be negative. Let $\left|x\right|$ denote the vector of absolute values of the components of x. It is easy to see that

(28) $$1^{⊺}\left|x\right|\geq 1,$$

and the equality is achieved if and only if x contains no negative components. Among all solutions x one can always find some for which $1^{⊺}\left|x\right|$ has the smallest possible value, and then

(29) $$\deg=1^{⊺}\left|x\right|-1$$

is a measure of contextuality. We refer to it as the *quasi-probability measure* of contextuality and denote it as ${\mathrm{CNT}}_{3}$, as in our previous publications.

For the third measure of contextuality, replace the equality in the definition of noncontextuality (8) with

(30) $$M\;x\leq v,\;x\geq 0\;\left(1^{⊺}x\leq 1\right),$$

where the inequalities are taken to hold component-wise. This inequality always has solutions, and among them there are some with the maximal value of $1^{⊺}x$. Then

(31) $$\deg=1-{\left(1^{⊺}x\right)}_{\max},$$

is a measure of contextuality, and it is called *contextual fraction, CNTF.*

The contextual fraction and the quasi-probability measures were first proposed by Abramsky and Brandenburger [4]. We later extended them to also apply to disturbed systems (see Ref. [2] for details). Note that the vector v representing a system, and therefore also the matrix M, are generally different for different measures of contextuality. For our three measures, they are the same for ${\mathrm{CNT}}_{2}$ and ${\mathrm{CNT}}_{3}$ but different for CNTF (for details, see Refs. [2,3]). Note also that when applied to undisturbed systems, ${\mathrm{CNT}}_{2}$ and CNTF produce the same contextuality values for complete couplings and reduced couplings; however, ${\mathrm{CNT}}_{3}$ values for complete and reduced couplings generally differ (which may be viewed as a weakness of this measure).

Each of the three measures, ${\mathrm{CNT}}_{2}$, ${\mathrm{CNT}}_{3}$, and CNTF, can be, at least in principle, applied to systems of any format. In particular, given a system R, each of them can be applied to all its level representations,

$$R\left[1\right],R\left[2\right],\ldots ,R\left[N\right],$$

to form their respective profiles.

## 6. Concatenated Systems

Contextuality profiles can be studied in many ways because, as all functions, they can be characterized in many ways. Moreover, as should be expected, their properties depend on the format of the systems we choose. This paper being introductory, we focus here on one aspect of contextuality profiles only: comparing our three measures of contextuality, ${\mathrm{CNT}}_{2}$, ${\mathrm{CNT}}_{3}$, and CNTF, on how fast the degree of contextuality tends to increase with its level. Let us explain what we mean by this.

Suppose a measure deg produces a profile that changes its value from $d_{n}$ to $d_{n+1}$ as one moves from level *n* to level n+1; and suppose that the corresponding values for another measure, $\deg^{\prime }$, are $d_{n}^{\prime }$ and $d_{n+1}^{\prime }$. Both measures are well-constructed, so

(32) $$d_{n+1}\geq d_{n},d_{n+1}^{\prime }\geq d_{n}^{\prime }.$$

However, it would not be informative to directly compare the numerical values of $d_{n+1}-d_{n}$ and $d_{n+1}^{\prime }-d_{n}^{\prime }$ (unless one of these differences is zero). The two measures are on completely different scales, so we may be comparing meters to grams, or even worse, meters to decibels. The same reasoning applies, of course, to their ratios, differences of their cubes, or other measures of incrementation. We need to find a way to consider the increase from $d_{n}$ to $d_{n+1}$ and from $d_{n}^{\prime }$ to $d_{n+1}^{\prime }$ intrinsically, within their respective scales.

How can this be done? The increase from $d_{n}$ to $d_{n+1}$ occurs because the system $R\left[n+1\right]$ contains $\left(n+1\right)$-tuples of variables, in addition to the *k*-tuples of variables with k≤n contained in $R\left[n\right]$. The degree of contextuality brought in by these $\left(n+1\right)$-tuples somehow combines with the contextuality present in $R\left[n\right]$ to produce $d_{n+1}$. If we had a way of measuring the contextuality $\Delta_{n+1}$ brought in by these $\left(n+1\right)$-tuples only, then we would be able to compare $d_{n}+\Delta_{n+1}$ to $d_{n+1}$:

(1) $d_{n}+\Delta_{n+1}<d_{n+1}$ (superadditive increment);
(2) $d_{n}+\Delta_{n+1}=d_{n+1}$ (additive increment);
(3) $d_{n}+\Delta_{n+1}>d_{n+1}$ (subadditive increment);
(4) $d_{n}=d_{n+1}$ (plateau).

But is there a way to find $\Delta_{n+1}$ independently of $d_{n+1}$? We propose one such way as follows.

Consider two systems,(33)
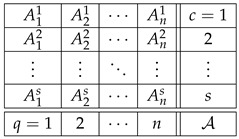

and
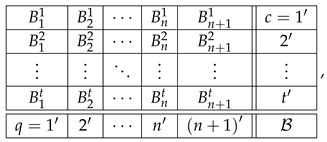

where some of the variables shown can be constants or empty cells. Let system A have a contextuality profile

(34) $$\begin{array}{ccccc} k & 1 & 2 & \cdots & n\\ \deg A\left[k\right] & 0 & d_{2} & \cdots & d_{n}\geq 0 \end{array}.$$

For system B, let us assume that its contextuality profile is

(35) $$\begin{array}{cccccc} k & 1 & 2 & \cdots & n & n+1\\ \deg B\left[k\right] & 0 & 0 & \cdots & 0 & \Delta_{n+1}\geq 0 \end{array}.$$

That is, this system is noncontextual at all levels except for the last one. Let us concatenate these two systems into a larger system as shown:(36)
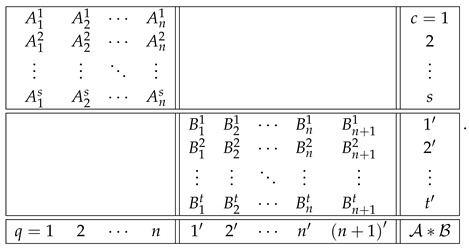

Note that the sets of both questions and contexts of the two subsystems are completely disjoint. This means that a coupling of A∗B can be constructed as separate couplings for A and for B, with their joint distribution defined arbitrarily (in particular, they can always be treated as independent events). In other words, for all k≤n, since $B\left[k\right]$ is noncontextual, the contextuality of $\left(A*B\right)\left[k\right]$ is determined by $A\left[k\right]$ alone. It is natural to expect then that, for all k≤n,

(37) $$\deg\left(A*B\right)\left[k\right]=\deg\left(A\right)\left[k\right].$$

This can even be added as a fourth requirement for a well-constructed measure of contextuality, in addition to the three requirements listed at the end of Section 4. Whether we do this or not, this property holds for all three measures, ${\mathrm{CNT}}_{2}$, ${\mathrm{CNT}}_{3}$, and CNTF. Consequently, for all of them we have

(38) $$\begin{array}{ccccc} k & 1 & 2 & \cdots & n\\ \deg A\left[k\right] & 0 & d_{2} & \cdots & d_{n}\\ \deg B\left[k\right] & 0 & 0 & \cdots & 0\\ \deg\left(A*B\right)\left[k\right] & 0 & d_{2} & \cdots & d_{n} \end{array}.$$

At the next, $\left(n+1\right)$st, level, we get

(39) $$\begin{array}{cccccc} k & 1 & 2 & \cdots & n & n+1\\ \deg A\left[k\right] & 0 & d_{2} & \cdots & d_{n} & d_{n}\\ \deg B\left[k\right] & 0 & 0 & \cdots & 0 & \Delta_{n+1}\\ \deg\left(A*B\right)\left[k\right] & 0 & d_{2} & \cdots & d_{n} & d_{n+1} \end{array}.$$

The reason $d_{n}$ repeats at level n+1 for $\deg A\left[k\right]$ is that $A\left[n+1\right]=A\left[n\right]$.

Clearly, we now have what we have aimed at: the possibility to compare $d_{n+1}$ and $d_{n}+\Delta_{n+1}$, in order to determine if the combination of $d_{n}$ and $\Delta_{n+1}$ by the measure deg is additive, superadditive, or subadditive (including the plateau case, $d_{n+1}=d_{n}$).

## 7. Contextuality Profiles for the Three Measures

**Figure 3:**
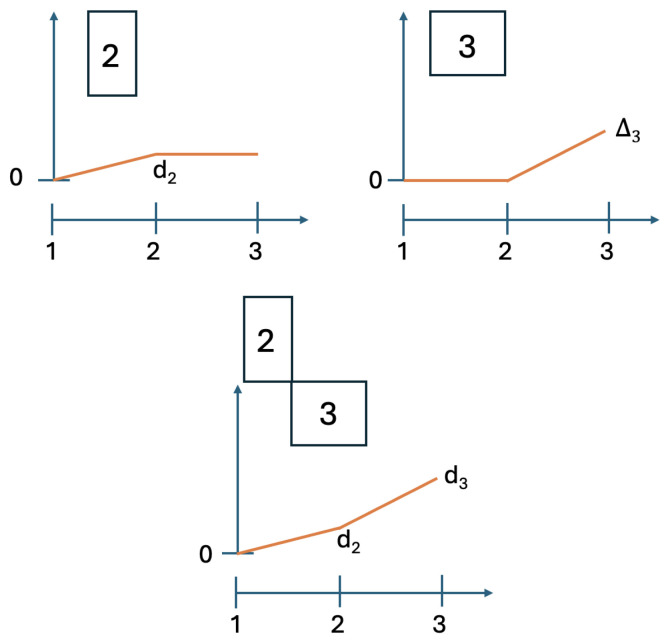
Contextuality profiles for the method of concatenated systems, n=2. The boxes represent the systems being concatenated, with the numbers in them indicating their final level of contextuality. Symbols attached to the curves indicate contextuality values.

**Figure 4:**
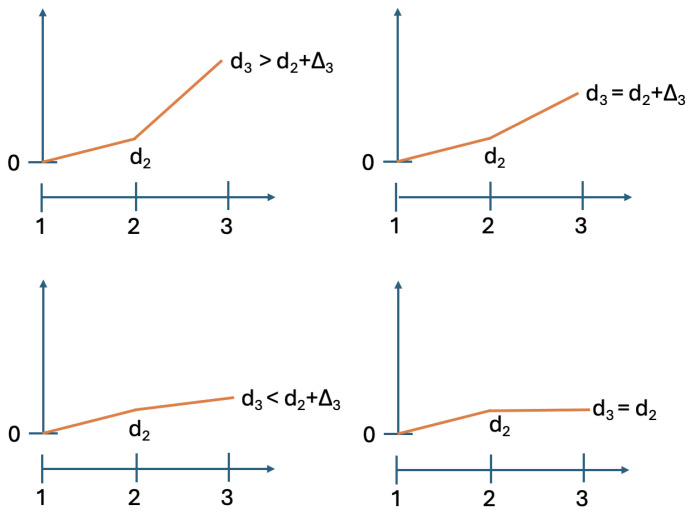
Four possible types of the contextuality profiles for concatenated systems (n=2): superadditive (**top left panel**), additive (**top right**), subadditive (**bottom left**), and, as the extreme case of subadditivity, plateau (**bottom right**).

We implement the method presented in the previous section using its simplest special case: with n=2. Figure 3 and Figure 4 illustrate the logic and the possible types of contextuality profiles for this special case.

We chose the formats for systems A and B as shown,(40)
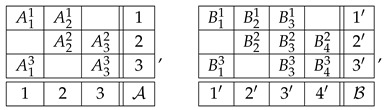

with all variables being dichotomous (say, ±1). The format of the concatenated system then acquires the form(41)
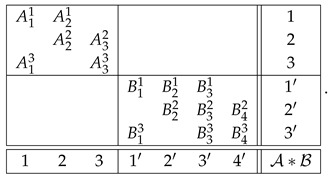

The systems we explored were obtained by specifying the joint distribution of the variables in the systems A and B. Each contextuality profile shown below has symbols attached to it, referring to the systems whose detailed specifications are given in Appendix A.

**Figure 5:**
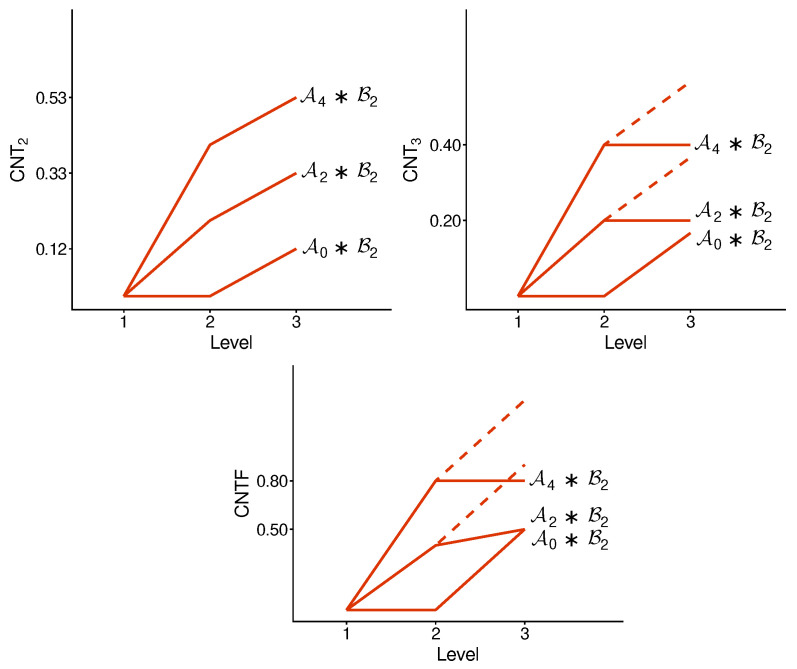
Contextuality profiles for a selection of undisturbed concatenated systems. Symbols A and B with indices refer to A- and B-subsystems, respectively (as specified in Appendix A). The dashed lines attached to each profile show the increment from $d_{2}$ to $d_{2}+\Delta_{3}$: if it is above the corresponding segment of the profile, we have subadditivity, and when the dashed line is not seen (coincides with the segment), we have additivity.

**Figure 6:**
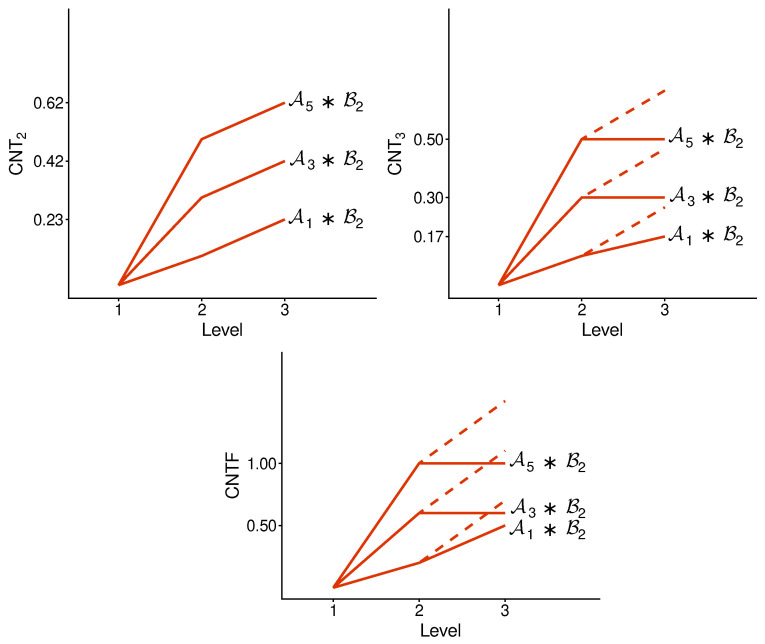
The same as in Figure 5, for another selection of the A-subsystems.

Figure 5 and Figure 6 show the contextuality profiles for a selection of undisturbed concatenated systems. We see that the measure ${\mathrm{CNT}}_{2}$ shows precise additivity, while both ${\mathrm{CNT}}_{3}$ and CNTF are subadditive. The subadditivity in these measures, especially in ${\mathrm{CNT}}_{3}$, is often extreme, resulting in a plateau in most cases shown.

**Figure 7:**
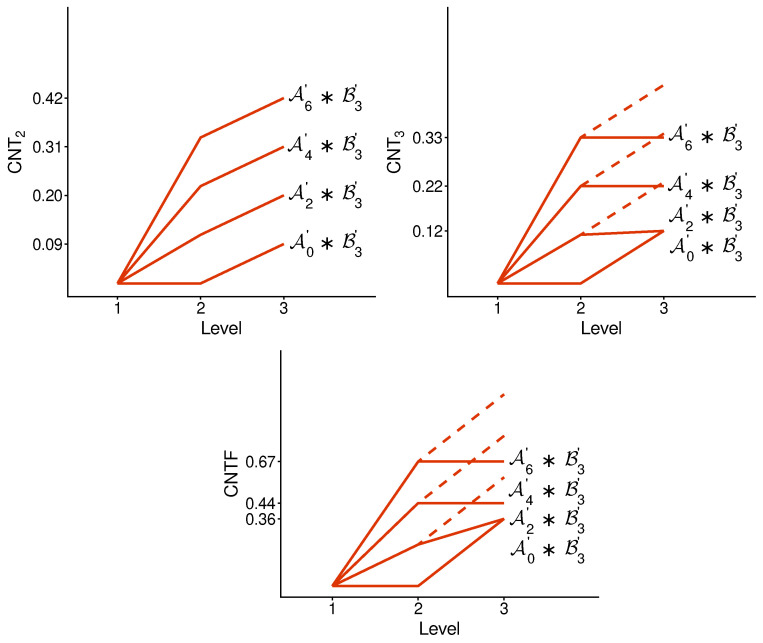
Contextuality profiles for a selection of disturbed A-subsystems concatenated with a disturbed subsystem $B_{3}$. The rest is the same as in Figure 5.

**Figure 8:**
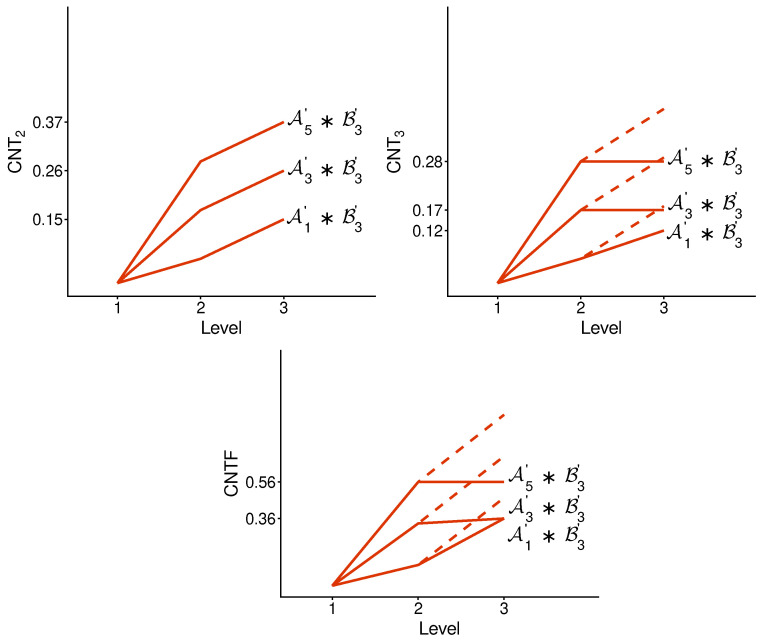
The same as in Figure 7 but for another selection of A-subsystems concatenated with a disturbed subsystem $B_{3}$.

There is no qualitative difference between the profiles of the undisturbed and disturbed concatenated systems. Figure 7 and Figure 8, for a selection of disturbed systems, exhibit the same pattern as in Figure 5 and Figure 6.

**Figure 9:**
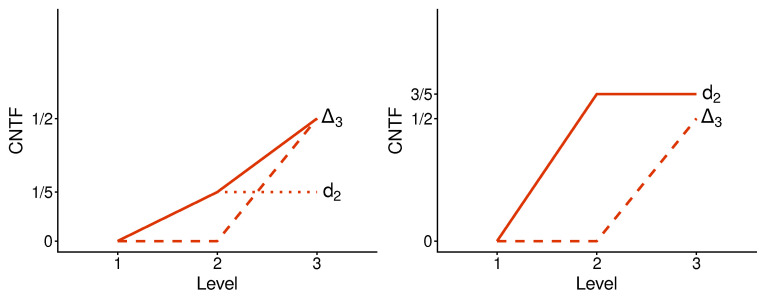
CNTF profiles for a selection of concatenated systems ($A_{1}*B_{2}$ left and $A_{3}*B_{2}$ right). The dashed lines represent the CNTF profiles for the systems’ B-parts. The dotted lines represent the CNTF profiles for the system’s A-parts (invisible if it coincides with a system’s profile).

**Figure 10:**
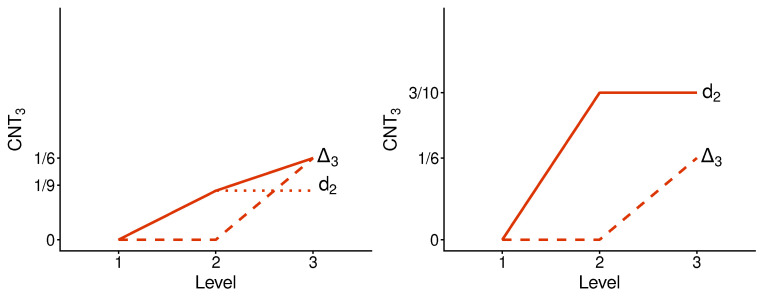
The same as in Figure 9 but for ${\mathrm{CNT}}_{3}$.

Now that the subadditivity of the contextuality profiles for ${\mathrm{CNT}}_{3}$ and CNTF has been observed, can we determine its cause? It turns out we can. Figure 9 and Figure 10 exhibit the contextuality profiles for ${\mathrm{CNT}}_{3}$ and CNTF with the superimposed profiles of the individual A- and B-subsystems. One can see that $d_{3}$ coincides with $\Delta_{3}$ if the latter exceeds $d_{2}$; otherwise, $d_{3}$ remains on the level of $d_{2}$. In other words, for both ${\mathrm{CNT}}_{3}$ and CNTF profiles shown, we have the rule of maximum:

(42) $$d_{3}=\max\left(d_{2},\Delta_{3}\right).$$

The subadditivity therefore is the consequence of

$$\max\left(d_{2},\Delta_{3}\right)\leq d_{2}+\Delta_{3}.$$

Table 1 and Table 2 provide an illustration of the addition rule for ${\mathrm{CNT}}_{2}$ and the rule of maximum for ${\mathrm{CNT}}_{3}$ and CNTF using larger selections of subsystems A and B than in our figures.

The results presented here are just a fraction of the systems we explored for this work: 6×49 undisturbed A-B pairs and 125×49 disturbed A-B pairs (with many different subsystems producing identical profiles). The contextuality curves we had to leave out in order not to clutter the graphs and tables or multiply their number conform to the same pattern: ${\mathrm{CNT}}_{2}$ is always additive, and the measures ${\mathrm{CNT}}_{3}$ and CNTF are subadditive because they conform to the rule of maximum.

With these regularities being established as inductive generalizations, we can look for their analytic justification. Although this is not essential for this paper, whose purpose is to introduce and demonstrate the usefulness of the concept of a contextuality profile for the discovery of regularities, we outline these analytic arguments below.

## 8. Outlines of the Proofs

For ${\mathrm{CNT}}_{2}$, since B is noncontextual at level 2, the value of $d_{2}$ is the $L_{1}$-distance between the system A and the level-2 noncontextuality polytope. The system and the polytope are defined in the space spanned by the axes representing all pairwise probabilities. The value of $\Delta_{3}$ is the $L_{1}$-distance between the system B and the level-3 noncontextuality polytope. Because B is noncontextual at level 2, this distance is entirely within the space spanned by the axes representing all triple probabilities. In the system A*B, the axes of these two spaces, of the pairwise and of the triple probabilities, are combined as mutually orthogonal subspaces. By the nature of $L_{1}$, therefore, the overall distance in this combined space is the sum of the two subspace distances. If instead of the $L_{1}$-distance we chose an $L_{p}$-distance with p>1, the overall distance would have satisfied

(43) $$d_{3}^{p}=d_{2}^{p}+\Delta_{3}^{p}.$$

The argument establishing the rule of maximum is essentially the same for ${\mathrm{CNT}}_{3}$ and CNTF. Let us present the details for the latter. With reference to (30) and (31), let $M_{A}$ be the Boolean incidence matrix for system A, let $x_{A}=\left(\alpha_{1},\ldots ,\alpha_{K}\right)$ be a vector of probabilities assigned to the *K* combinations of values of the variables in A, and let $v_{A}$ be the vector of probabilities. We define analogously $M_{B}$, $x_{B}=\left(\beta_{1},\ldots ,\beta_{L}\right)$, and $v_{B}$ for system B, and $M_{AB}$, $x_{AB}=\left(\gamma_{11},\ldots ,\gamma_{KL}\right)$, and $v_{AB}$ for system A∗B. Here, $\gamma_{ij}$ is assigned to the concatenation of the *i*th combination of values in A and the *j*th combination of values in B; and

(44) $$v_{AB}=\left(\begin{array}{c} v_{A}\\ v_{B} \end{array}\right).$$

In matrix $M_{A}$, let $a_{is}$$\left(i=1,\ldots ,K\right)$ be the entries of the row corresponding to the *s*th element $v_{A}^{\left(s\right)}$ of $v_{A}$, and let $b_{jt}$$\left(j=1,\ldots ,L\right)$ be the entries of the row corresponding to the *t*th element $v_{B}^{\left(t\right)}$ of $v_{B}$. For matrix $M_{AB}$, let $c_{ijs}$ and $c_{ijt}$ be the entries of the rows corresponding, respectively, to $v_{A}^{\left(s\right)}$ and to $v_{B}^{\left(t\right)}$ in (44). We have

(45) $$M_{A}x_{A}=\left\{\begin{array}{c} \vdots \\ \sum_{i=1}^{K}a_{is}\alpha_{i}\\ \vdots \end{array}\right.\;\leq \left\{\begin{array}{c} \vdots \\ v_{A}^{\left(s\right)}\\ \vdots \end{array}\right.=v_{A},$$

(46) $$M_{B}x_{B}=\left\{\begin{array}{c} \vdots \\ \sum_{j=1}^{L}b_{jt}\beta_{j}\\ \vdots \end{array}\right.\;\leq \left\{\begin{array}{c} \vdots \\ v_{B}^{\left(t\right)}\\ \vdots \end{array}\right.=v_{B}$$

and

(47) $$M_{AB}x_{AB}=\left\{\begin{array}{c} \vdots \\ \sum_{i=1}^{K}\sum_{j=1}^{L}c_{ijs}\gamma_{ij}\\ \vdots \\ \sum_{j=1}^{L}\sum_{i=1}^{K}c_{ijt}\gamma_{ij}\\ \vdots \end{array}\right.\;\leq \left\{\begin{array}{c} \vdots \\ v_{A}^{\left(s\right)}\\ \vdots \\ v_{B}^{\left(t\right)}\\ \vdots \end{array}\right.=\left(\begin{array}{c} v_{A}\\ v_{B} \end{array}\right).$$

Due to the structure of a concatenated system,

(48) $$c_{ijs}=a_{is}$$

irrespective of j=1,…,L, and

(49) $$c_{ijt}=b_{jt}$$

irrespective of i=1,…,K. Therefore, the inequality for system A*B can be written as

(50) $$M_{AB}x_{AB}=\left\{\begin{array}{c} \vdots \\ \sum_{i=1}^{K}a_{is}\sum_{j=1}^{L}\gamma_{ij}\\ \vdots \\ \sum_{j=1}^{L}b_{jt}\sum_{i=1}^{K}\gamma_{ij}\\ \vdots \end{array}\right.\;\leq \left\{\begin{array}{c} \vdots \\ v_{A}^{\left(s\right)}\\ \vdots \\ v_{B}^{\left(t\right)}\\ \vdots \end{array}\right.=\left(\begin{array}{c} v_{A}\\ v_{B} \end{array}\right).$$

Let us show now that

(51) $${\left(1^{⊺}x_{AB}\right)}_{\max}\leq \min\left({\left(1^{⊺}x_{A}\right)}_{\max},{\left(1^{⊺}x_{B}\right)}_{\max}\right).$$

Indeed, if we had, e.g., ${\left(1^{⊺}x_{AB}\right)}_{\max}>{\left(1^{⊺}x_{B}\right)}_{\max}$, then we could redefine the values of $x_{B}$ as

(52) $$\beta_{j}=\sum_{i=1}^{K}\gamma_{ij},j=1,\ldots ,L,$$

and, by substituting in (46), obtain a coupling of B with a greater value of $1^{⊺}x_{B}$ than ${\left(1^{⊺}x_{B}\right)}_{\max}$. The inequality (46) with the redefined vector will be preserved because it holds in (50).

It is also true that

(53) $${\left(1^{⊺}x_{AB}\right)}_{\max}\geq \min\left({\left(1^{⊺}x_{A}\right)}_{\max},{\left(1^{⊺}x_{B}\right)}_{\max}\right),$$

because if we had, e.g., ${\left(1^{⊺}x_{AB}\right)}_{\max}<{\left(1^{⊺}x_{B}\right)}_{\max}\leq {\left(1^{⊺}x_{A}\right)}_{\max}$, then we could redefine the values of $x_{AB}$ as

(54) $$\gamma_{ij}=\left\{\begin{array}{ccc} \beta_{j} & \mathrm{if} & i=1,\\ 0 & \mathrm{if} & i>1, \end{array}\right.$$

and obtain thereby a coupling of A∗B with a greater value of $1^{⊺}x_{AB}$ than ${\left(1^{⊺}x_{B}\right)}_{\max}$. The inequality in (50) with the redefined vector will be preserved because it holds in (46). The conjunction of (51) and (53) yields the rule of maximum, (42), because

$$\mathrm{CNTF}=1-{\left(1^{⊺}x\right)}_{\max}.$$

For ${\mathrm{CNT}}_{3}$, with reference to (27) and (29), we show that

(55) $${\left(1^{⊺}\left|x_{AB}\right|\right)}_{\max}\geq \min\left({\left(1^{⊺}\left|x_{A}\right|\right)}_{\max},{\left(1^{⊺}\left|x_{B}\right|\right)}_{\max}\right),$$

because if, e.g., ${\left(1^{⊺}\left|x_{AB}\right|\right)}_{\max}<{\left(1^{⊺}\left|x_{B}\right|\right)}_{\max}$, one could redefine the values of $x_{AB}$ as in (54), and achieve an increase in $1^{⊺}\left|x_{AB}\right|$. By the same argument as above, the equality $M_{AB}x_{AB}=v_{AB}$ will be preserved because $M_{B}x_{B}=v_{B}$.

Also,

(56) $${\left(1^{⊺}\left|x_{AB}\right|\right)}_{\max}\leq \min\left({\left(1^{⊺}\left|x_{A}\right|\right)}_{\max},{\left(1^{⊺}\left|x_{B}\right|\right)}_{\max}\right),$$

because if, e.g., ${\left(1^{⊺}\left|x_{AB}\right|\right)}_{\max}>{\left(1^{⊺}\left|x_{B}\right|\right)}_{\max}\geq {\left(1^{⊺}\left|x_{A}\right|\right)}_{\max}$, one could redefine the values of $x_{B}$ as in (52), and achieve an increase in $1^{⊺}\left|x_{B}\right|$.

The equality $M_{B}x_{B}=v_{B}$ will be presented because $M_{AB}x_{AB}=v_{AB}$. (Note that the dimensions and entries of the vectors and matrices are different for ${\mathrm{CNT}}_{3}$ and CNTF; see Ref. [3]).

## 9. Hypercyclic Systems

Hypercyclic systems were introduced in Ref. [6] as a set of systems that are both highly structured and sufficiently diverse to form testing grounds for contextuality research. The subsystem A in our concatenated systems is a cyclic system of rank 3 (a special case of a hypercyclic system), and the subsystem B is a hypercyclic system of order 3 and rank 4 but without a last row (so abridged to speed up execution of the linear programs). The order of a hypercyclic system is the number of variables in each context; the rank is the number of the system’s contexts (which is the same as the number of questions). The variables in each row are cyclically shifted clockwise with respect to the previous row.

**Figure 11:**
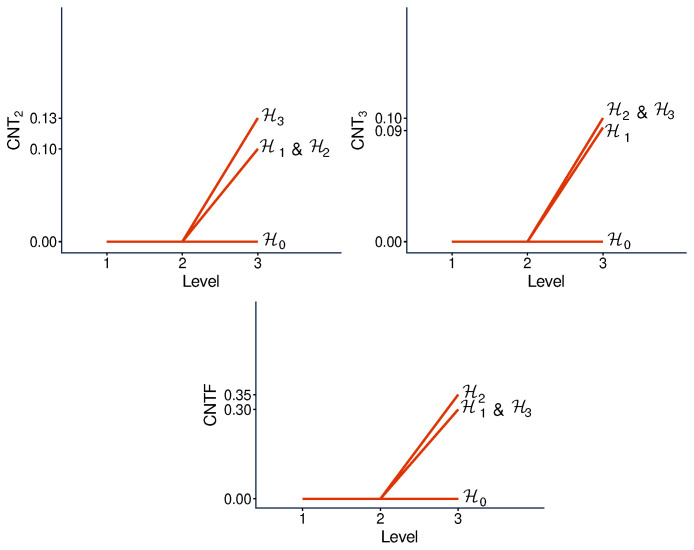
Contextuality profiles for a selection of undisturbed hypercyclic systems of order 3 and rank 4.

**Figure 12:**
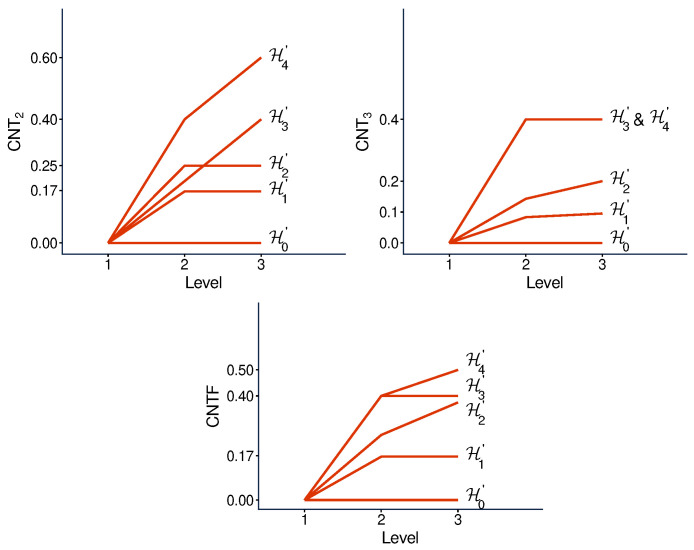
Contextuality profiles for a selection of disturbed hypercyclic systems of order 3 and rank 4.

Figure 11 and Figure 12 present contextuality profiles for a selection of hypercyclic systems of order 3 and rank 4:(57)
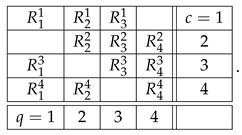


Figure 11 has the property common to all undisturbed hypercyclic systems: they are noncontextual at all but the final level (in our case, level 3). Some disturbed hypercyclic systems have this property too, but it does not hold generally.

**Figure 13:**
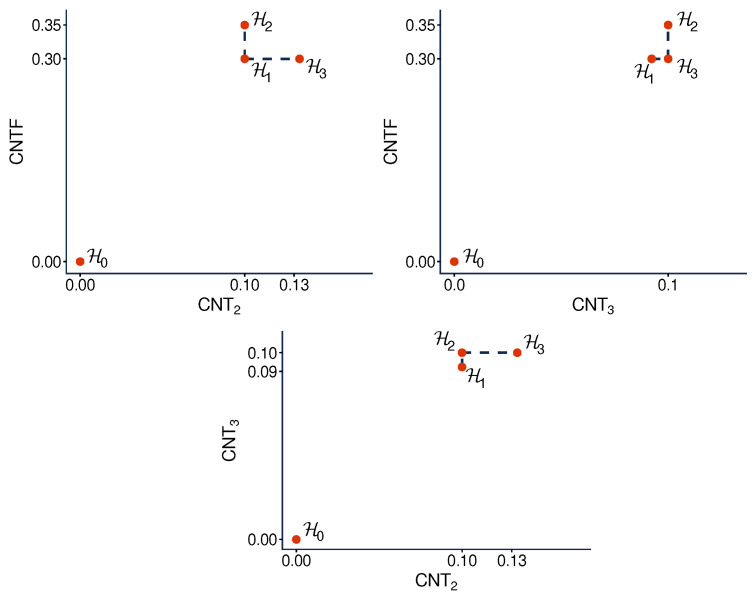
Relationship between the overall contextuality degrees generated by two contextuality measures applied to our selection of undisturbed hypercyclic systems. The horizontal lines show that the abscissa measure cannot be a function of the ordinate one; the vertical lines show that the ordinate measure cannot be a function of the abscissa one.

However, this property is not why we consider the hypercyclic systems here. In this paper they serve another purpose. We know from Ref. [6] that none of the three measures of contextuality we studied, ${\mathrm{CNT}}_{2}$, ${\mathrm{CNT}}_{3}$, and CNTF, is a function of any other one of them when considered across different systems. Specifically, it was shown that, for any ordered pair of these contextuality measures, e.g., $\left(\mathrm{CNTF},{\mathrm{CNT}}_{2}\right)$, one can find two hypercyclic systems such that the first measure changes from one of them to another, while the second measure remains constant. For our selection of undisturbed hypercyclic systems, this is shown in Figure 13. The interpretation of this observation is that, unlike, say, CNTF and $\log\left(\mathrm{CNTF}\right)$, the three measures ${\mathrm{CNT}}_{2}$, CNTF, and ${\mathrm{CNT}}_{3}$ reflect pairwise distinct aspects of contextuality.

**Figure 14:**
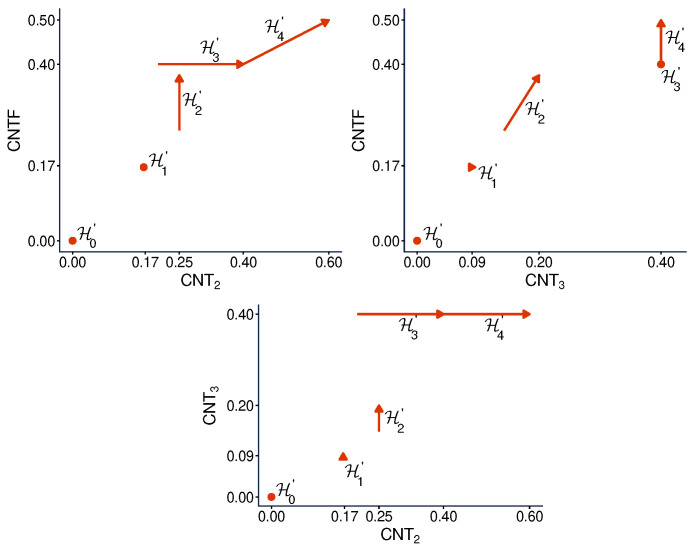
Relationship between the segments of contextuality profiles (between levels 2 and 3, as indicated by arrows) generated by two measures applied to the same disturbed hypercyclic system. The horizontal lines show that the abscissa profile cannot be a function of the ordinate one; the vertical lines show that the ordinate profile cannot be a function of the abscissa one.

The question we pose in this paper is whether the same is true for the contextual profiles generated by the three measures for one and the same system. Figure 14 tells us that this is indeed the case: for any ordered pair of our three measures, one can find a system such that the first measure changes between levels 2 and 3 while the second measure remains constant.

## 10. Concluding Discussion

We have introduced a new notion, that of a contextuality profile of a system, and investigated some of its basic properties. We compared the contextuality profiles of three well-constructed measures, ${\mathrm{CNT}}_{2}$, CNTF, and ${\mathrm{CNT}}_{3}$, using the method of concatenated systems. We established that ${\mathrm{CNT}}_{2}$ profiles are additive, while CNTF and ${\mathrm{CNT}}_{3}$ profiles are subadditive because they conform to the rule of maximum. We have also established that none of these three measures is a function of any other, not only across different systems (which has been known previously), but also within a system taken at different levels.

**Figure 15:**
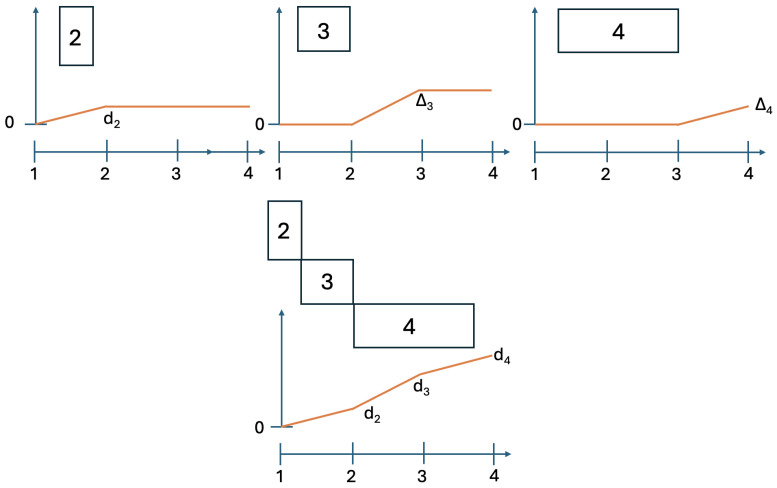
Contextuality profiles for a triple concatenation of systems. The boxes represent the systems being concatenated, with the numbers in them indicating their final level of contextuality.

Note that concatenation can be used recursively, creating combinations of three and more systems, as shown in Figure 15. For concatenations of $A_{1},\ldots ,A_{k}$, the arguments for the additivity of ${\mathrm{CNT}}_{2}$ and the maximum rule for CNTF and ${\mathrm{CNT}}_{3}$ can be recursively applied to show that

(58) $${\mathrm{CNT}}_{2}=\sum_{i=2}^{k}\Delta_{i},{\mathrm{CNT}}_{3}=\max\left(\Delta_{2}^{\prime },\ldots ,\Delta_{k}^{\prime }\right),\mathrm{CNTF}=\max\left(\Delta_{2}^{\prime \prime },\ldots ,\Delta_{k}^{\prime \prime }\right),$$

where we replaced $d_{2}$ with $\Delta_{2}$ for uniformity and added primes to emphasize that the value of $\Delta_{i}$ is measure-specific.

This being only a concept paper, it leaves many questions unanswered. From a mathematical point of view, much remains to be investigated analytically regarding the properties of the contextuality profiles. However, aside from the intrinsic mathematical interest, the main substantive question is that of applicability: do the contextuality profiles tell us something about other, independently defined properties of the empirical entities described by the systems? Could, e.g., the resource-theoretical aspects of the systems [7] be better understood if we relate them to various aspects of contextuality profiles rather than to the overall degree of contextuality only? Another possible application was pointed out to us by Paweł Kurzyński [8]: he suggested comparing the contextuality profiles of the two well-known quantum systems, the Greenberger–Horne–Zeilinger system (GHZ, [9]) and the W-state one [10]. Both are known to be triple-entangled, but only the W-state system is also pairwise-entangled. So it is of interest to see if this difference is reflected in the contextuality profiles. It does not have to be, because the relationship between quantum entanglement and contextuality (including nonlocality as its special case) is not one-to-one. An entangled system may very well be noncontextual, and it is only an assumption based on an empirical generalization that any unentangled system is noncontextual.

The tripartite W-state is

(59) $$|W\rangle =\frac{1}{\sqrt{3}}\left(|001\rangle +|010\rangle +|100\rangle \right),$$

and the W-state system of random variables has the format(60)
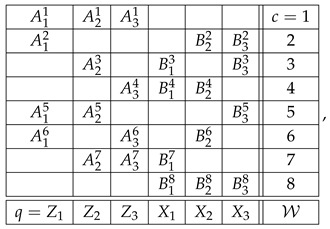

where $Z_{i}$ and $X_{i}$ denote the dichotomous measurements on the *i*th particle along the *z*-axis and *x*-axis, respectively. For convenience, we write $A_{i}^{c}$ and $B_{i}^{c}$ in place of $R_{Z_{i}}^{c}$ and $R_{X_{i}}^{c}$. The distributions of the variables in each context of W, as derived from (59), are shown in Appendix B. The contextuality of system W at level 3 was established in Ref. [11], and our computations (using reduced couplings because the system is undisturbed) show that at this level CNT2=18, CNT3=12 and CNTF=12. Ref. [12] claims to have established that the W-state system is also contextual at level 2, aligning with the fact that its components are pairwise entangled. However, this claim is not supported by our computations, showing that W is noncontextual at level 2. Appendix B presents the coupling of the system (Table A8) from which all the pairwise probabilities in W (Table A7) are obtained as marginals. This is a reduced coupling

(61) $$\left(S_{1},S_{2},S_{3},T_{1},T_{2},T_{3}\right)$$

such that

(62) $$\left(S_{i},S_{j}\right)\overset{d}{=}\left(A_{i}^{c},A_{j}^{c}\right),\left(S_{i},T_{j}\right)\overset{d}{=}\left(A_{i}^{c},B_{j}^{c}\right),\left(T_{i},T_{j}\right)\overset{d}{=}\left(B_{i}^{c},B_{j}^{c}\right)$$

for all $i,j\in \left\{1,2,3\right\}$ and any context *c*.

The tripartite GHZ-state is

(63) $$|GHZ\rangle =\frac{1}{\sqrt{2}}\left(|y_{+}y_{+}y_{+}\rangle +|y_{-}y_{-}y_{-}\rangle \right),$$

and the GHZ system of random variables has the same format as W. All its properties, however, can be established in its abridged version(64)
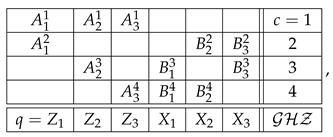

with the distributions shown in Appendix B. The contextuality of this system at level 3 was also established in Ref. [11], and our computations, using reduced couplings, yield CNT2=14, ${\mathrm{CNT}}_{3}=1$, and CNTF=1. The noncontextuality of the GHZ system at level 2 is obvious because the random variables at this level are stochastically independent in every context (see Table A7 in Appendix B).

As we see, the contextuality profiles of the systems GHZ and W are qualitatively the same (0 at level 2 and some positive value at level 3), in spite of the fact that a physical system in the $|W\rangle$ state is pairwise entangled while a physical system in the $|GHZ\rangle$ state is not. More work is needed in search of more informative applications.

## Figures and Tables

**Table 1:** Contextuality value $d_{3}$ of undisturbed concatenated systems for the measures ${\mathrm{CNT}}_{2}$, ${\mathrm{CNT}}_{3}$, and CNTF. The value of $d_{3}$ for $A_{i}*B_{j}$ is in the intersection of column $A_{i}$ and row $B_{j}$. The corresponding values of $d_{2}$ for $A_{i}$ and $\Delta_{3}$ for $B_{j}$ are shown, respectively, just under $A_{i}$ and just to the right of $B_{j}$. Observe that for ${\mathrm{CNT}}_{2}$, $d_{3}$ is the sum of the corresponding values of $d_{2}$ and $\Delta_{3}$; and for both ${\mathrm{CNT}}_{3}$ and CNTF, $d_{3}$ is the larger of the corresponding values of $d_{2}$ and $\Delta_{3}$. (Note that for the A-subsystems, ${\mathrm{CNT}}_{2}={\mathrm{CNT}}_{3}=\frac{1}{2}\mathrm{CNTF}$, as it was previously established for all cyclic systems [5]).

${\mathrm{CNT}}_{2}$		$A_{0}$	$A_{1}$	$A_{2}$	$A_{3}$	$A_{4}$	$A_{5}$
		0	110	15	310	25	12
$B_{1}$	124	124	17120	29120	41120	53120	1324
$B_{2}$	18	18	940	1340	1740	2140	58
${\mathrm{CNT}}_{3}$		$A_{0}$	$A_{1}$	$A_{2}$	$A_{3}$	$A_{4}$	$A_{5}$
		0	110	15	310	25	12
$B_{1}$	118	118	110	15	310	25	12
$B_{2}$	16	16	16	15	310	25	12
CNTF		$A_{0}$	$A_{1}$	$A_{2}$	$A_{3}$	$A_{4}$	$A_{5}$
		0	15	25	35	45	1
$B_{1}$	16	16	15	25	35	45	1
$B_{2}$	12	12	12	12	35	45	1

**Table 2:** Contextuality value $d_{3}$ of disturbed concatenated systems for the measures ${\mathrm{CNT}}_{2}$, ${\mathrm{CNT}}_{3}$, and CNTF. The rest as in Table 1.

${\mathrm{CNT}}_{2}$		$A_{0}^{\prime }$	$A_{1}^{\prime }$	$A_{2}^{\prime }$	$A_{3}^{\prime }$	$A_{4}^{\prime }$	$A_{5}^{\prime }$	$A_{6}^{\prime }$
		0	118	19	16	29	518	39
$B_{1}^{\prime }$	1100	1100	59900	109900	159900	209900	259900	309900
$B_{2}^{\prime }$	5100	5100	19180	29180	1360	49180	59180	23180
$B_{3}^{\prime }$	9100	9100	131900	181900	77300	281900	331900	127300
${\mathrm{CNT}}_{3}$		$A_{0}^{\prime }$	$A_{1}^{\prime }$	$A_{2}^{\prime }$	$A_{3}^{\prime }$	$A_{4}^{\prime }$	$A_{5}^{\prime }$	$A_{6}^{\prime }$
		0	118	19	16	29	518	39
$B_{1}^{\prime }$	2150	2150	118	19	16	29	518	39
$B_{2}^{\prime }$	115	115	115	19	16	29	518	39
$B_{3}^{\prime }$	325	325	325	325	16	29	518	39
CNTF		$A_{0}^{\prime }$	$A_{1}^{\prime }$	$A_{2}^{\prime }$	$A_{3}^{\prime }$	$A_{4}^{\prime }$	$A_{5}^{\prime }$	$A_{6}^{\prime }$
		0	19	29	13	49	59	23
$B_{1}^{\prime }$	125	125	19	29	13	49	59	23
$B_{2}^{\prime }$	15	15	15	29	13	49	59	23
$B_{3}^{\prime }$	925	925	925	925	925	49	59	23

## Data Availability

No data was used for this research. The code for computing the measures and for the numerical exploration can be found at https://github.com/herulor/ContextualityMeasures/ (accessed on 23 March 2026).

## Appendix A

Table A1, Table A2, Table A3, Table A4, Table A5 and Table A6 show the distributions of all the systems mentioned in the main text. Disturbed systems are indicated by primes ($A_{1}^{\prime }$, $B_{2}^{\prime }$, $H_{0}^{\prime }$, etc.).

**Table A1:** Distributions of the variables in the selection of the undisturbed A-subsystems used. All variables are dichotomous (±1). Each number shows the probability with which the corresponding variable(s) equal 1. Thus, $\frac{a\;b}{c}$ in the second row means that $P\left[R_{2}^{2}=1\right]=a$, $P\left[R_{3}^{2}=1\right]=b$, and $P\left[R_{2}^{2}=R_{3}^{2}=1\right]=c$.

	$A_{0}$	$A_{1}$	$A_{2}$	$A_{3}$	$A_{4}$	$A_{5}$
$\begin{array}{c} \begin{array}{cc} R_{1}^{1} & R_{2}^{1} \end{array}\\ R_{1}^{1}=R_{2}^{1} \end{array}$	$\begin{array}{c} \begin{array}{cc} 0.5 & 0.5 \end{array}\\ 0 \end{array}$	$\begin{array}{c} \begin{array}{cc} 0.5 & 0.5 \end{array}\\ 0 \end{array}$	$\begin{array}{c} \begin{array}{cc} 0.5 & 0.5 \end{array}\\ 0 \end{array}$	$\begin{array}{c} \begin{array}{cc} 0.5 & 0.5 \end{array}\\ 0 \end{array}$	$\begin{array}{c} \begin{array}{cc} 0.5 & 0.5 \end{array}\\ 0 \end{array}$	$\begin{array}{c} \begin{array}{cc} 0.5 & 0.5 \end{array}\\ 0 \end{array}$
$\begin{array}{c} \begin{array}{cc} R_{2}^{2} & R_{3}^{2} \end{array}\\ R_{2}^{2}=R_{3}^{2} \end{array}$	$\begin{array}{c} \begin{array}{cc} 0.5 & 0.5 \end{array}\\ 0 \end{array}$	$\begin{array}{c} \begin{array}{cc} 0.5 & 0.5 \end{array}\\ 0 \end{array}$	$\begin{array}{c} \begin{array}{cc} 0.5 & 0.5 \end{array}\\ 0 \end{array}$	$\begin{array}{c} \begin{array}{cc} 0.5 & 0.5 \end{array}\\ 0 \end{array}$	$\begin{array}{c} \begin{array}{cc} 0.5 & 0.5 \end{array}\\ 0 \end{array}$	$\begin{array}{c} \begin{array}{cc} 0.5 & 0.5 \end{array}\\ 0 \end{array}$
$\begin{array}{c} \begin{array}{cc} R_{3}^{3} & R_{4}^{3} \end{array}\\ R_{3}^{3}=R_{4}^{3} \end{array}$	$\begin{array}{c} \begin{array}{cc} 0.5 & 0.5 \end{array}\\ 0.5 \end{array}$	$\begin{array}{c} \begin{array}{cc} 0.5 & 0.5 \end{array}\\ 0.4 \end{array}$	$\begin{array}{c} \begin{array}{cc} 0.5 & 0.5 \end{array}\\ 0.3 \end{array}$	$\begin{array}{c} \begin{array}{cc} 0.5 & 0.5 \end{array}\\ 0.2 \end{array}$	$\begin{array}{c} \begin{array}{cc} 0.5 & 0.5 \end{array}\\ 0.1 \end{array}$	$\begin{array}{c} \begin{array}{cc} 0.5 & 0.5 \end{array}\\ 0 \end{array}$

**Table A2:** The same as Table A1 but for the selection of the disturbed A-subsystems used.

	$A_{0}^{\prime }$	$A_{1}^{\prime }$	$A_{2}^{\prime }$	$A_{3}^{\prime }$	$A_{4}^{\prime }$	$A_{5}^{\prime }$	$A_{6}^{\prime }$
$\begin{array}{c} \begin{array}{cc} R_{1}^{1} & R_{2}^{1} \end{array}\\ R_{1}^{1}=R_{2}^{1} \end{array}$	49590	49590	49590	49590	49590	49590	49590
$\begin{array}{c} \begin{array}{cc} R_{2}^{2} & R_{3}^{2} \end{array}\\ R_{2}^{2}=R_{3}^{2} \end{array}$	495919	4959118	49590	49590	49590	49590	49590
$\begin{array}{c} \begin{array}{cc} R_{3}^{3} & R_{4}^{3} \end{array}\\ R_{3}^{3}=R_{4}^{3} \end{array}$	495929	495929	495929	4959318	495919	4959118	49590

**Table A3:** Distributions of the variables in the selection of the undisturbed B-subsystems used. As in Table A1, each number shows the probability with which the corresponding variable(s) equal 1. Thus, $\frac{\frac{a\;b\;c}{d\;e\;f}}{g}$ in the second row means that $P\left[R_{2}^{2}=1\right]=a$, $P\left[R_{2}^{2}=R_{3}^{2}=1\right]=d$, $P\left[R_{2}^{2}=R_{3}^{2}=R_{4}^{2}=1\right]=g$, etc.

	$B_{1}$	$B_{2}$
$\begin{array}{c} \begin{array}{ccc} R_{1}^{1} & R_{2}^{1} & R_{3}^{1} \end{array}\\ \begin{array}{ccc} R_{1}^{1}=R_{2}^{1} & R_{2}^{1}=R_{3}^{1} & R_{1}^{1}=R_{3}^{1} \end{array}\\ R_{1}^{1}=R_{1}^{1}=R_{3}^{1} \end{array}$	12121214141418	12121214141418
$\begin{array}{c} \begin{array}{ccc} R_{2}^{2} & R_{3}^{2} & R_{4}^{2} \end{array}\\ \begin{array}{ccc} R_{2}^{2}=R_{3}^{2} & R_{3}^{2}=R_{4}^{2} & R_{2}^{2}=R_{4}^{2} \end{array}\\ R_{2}^{2}=R_{3}^{2}=R_{4}^{2} \end{array}$	1212121414140	12121214141416
$\begin{array}{c} \begin{array}{ccc} R_{3}^{3} & R_{4}^{3} & R_{1}^{3} \end{array}\\ \begin{array}{ccc} R_{3}^{3}=R_{4}^{3} & R_{4}^{3}=R_{1}^{3} & R_{3}^{3}=R_{1}^{3} \end{array}\\ R_{3}^{3}=R_{4}^{3}=R_{1}^{3} \end{array}$	12121214141414	1212121414140

**Table A4:** The same as Table A3, but for the selection of the disturbed B-subsystems used.

	$B_{1}^{\prime }$	$B_{2}^{\prime }$	$B_{3}^{\prime }$
$\begin{array}{c} \begin{array}{ccc} R_{1}^{1} & R_{2}^{1} & R_{3}^{1} \end{array}\\ \begin{array}{ccc} R_{1}^{1}=R_{2}^{1} & R_{2}^{1}=R_{3}^{1} & R_{1}^{1}=R_{3}^{1} \end{array}\\ R_{1}^{1}=R_{1}^{1}=R_{3}^{1} \end{array}$	13251212251350625625325	13251212251350625625325	13251212251350625625325
$\begin{array}{c} \begin{array}{ccc} R_{2}^{2} & R_{3}^{2} & R_{4}^{2} \end{array}\\ \begin{array}{ccc} R_{2}^{2}=R_{3}^{2} & R_{3}^{2}=R_{4}^{2} & R_{2}^{2}=R_{4}^{2} \end{array}\\ R_{2}^{2}=R_{3}^{2}=R_{4}^{2} \end{array}$	132512122513506256250	132512122513506256250	132512122513506256250
$\begin{array}{c} \begin{array}{ccc} R_{3}^{3} & R_{4}^{3} & R_{1}^{3} \end{array}\\ \begin{array}{ccc} R_{3}^{3}=R_{4}^{3} & R_{4}^{3}=R_{1}^{3} & R_{3}^{3}=R_{1}^{3} \end{array}\\ R_{3}^{3}=R_{4}^{3}=R_{1}^{3} \end{array}$	13251212251350625625225	13251212251350625625125	132512122513506256250

**Table A5:** Distributions in the selection of the undisturbed hypercyclic systems used. The format is the same as in Table A3.

	$H_{0}$	$H_{1}$	$H_{2}$	$H_{3}$
$\begin{array}{c} \begin{array}{ccc} R_{1}^{1} & R_{2}^{1} & R_{3}^{1} \end{array}\\ \begin{array}{ccc} R_{1}^{1}=R_{2}^{1} & R_{2}^{1}=R_{3}^{1} & R_{1}^{1}=R_{3}^{1} \end{array}\\ R_{1}^{1}=R_{1}^{1}=R_{3}^{1} \end{array}$	$\begin{array}{c} \begin{array}{ccc} 1\;/\;2 & 1\;/\;2 & 1\;/\;2 \end{array}\\ \begin{array}{ccc} 1\;/\;4 & 1\;/\;4 & 1\;/\;4 \end{array}\\ 1\;/\;5 \end{array}$	$\begin{array}{c} \begin{array}{ccc} 1\;/\;2 & 1\;/\;2 & 1\;/\;2 \end{array}\\ \begin{array}{ccc} 1\;/\;4 & 1\;/\;4 & 1\;/\;4 \end{array}\\ 1\;/\;5 \end{array}$	$\begin{array}{c} \begin{array}{ccc} 1\;/\;2 & 1\;/\;2 & 1\;/\;2 \end{array}\\ \begin{array}{ccc} 1\;/\;4 & 1\;/\;4 & 1\;/\;4 \end{array}\\ 1\;/\;4 \end{array}$	$\begin{array}{c} \begin{array}{ccc} 1\;/\;2 & 1\;/\;2 & 1\;/\;2 \end{array}\\ \begin{array}{ccc} 1\;/\;4 & 1\;/\;4 & 1\;/\;4 \end{array}\\ 1\;/\;5 \end{array}$
$\begin{array}{c} \begin{array}{ccc} R_{2}^{2} & R_{3}^{2} & R_{4}^{2} \end{array}\\ \begin{array}{ccc} R_{2}^{2}=R_{3}^{2} & R_{3}^{2}=R_{4}^{2} & R_{2}^{2}=R_{4}^{2} \end{array}\\ R_{2}^{2}=R_{3}^{2}=R_{4}^{2} \end{array}$	$\begin{array}{c} \begin{array}{ccc} 1\;/\;2 & 1\;/\;2 & 1\;/\;2 \end{array}\\ \begin{array}{ccc} 1\;/\;4 & 1\;/\;4 & 1\;/\;4 \end{array}\\ 3\;/\;20 \end{array}$	$\begin{array}{c} \begin{array}{ccc} 1\;/\;2 & 1\;/\;2 & 1\;/\;2 \end{array}\\ \begin{array}{ccc} 1\;/\;4 & 1\;/\;4 & 1\;/\;4 \end{array}\\ 1\;/\;5 \end{array}$	$\begin{array}{c} \begin{array}{ccc} 1\;/\;2 & 1\;/\;2 & 1\;/\;2 \end{array}\\ \begin{array}{ccc} 1\;/\;4 & 1\;/\;4 & 1\;/\;4 \end{array}\\ 1\;/\;5 \end{array}$	$\begin{array}{c} \begin{array}{ccc} 1\;/\;2 & 1\;/\;2 & 1\;/\;2 \end{array}\\ \begin{array}{ccc} 1\;/\;4 & 1\;/\;4 & 1\;/\;4 \end{array}\\ 1\;/\;5 \end{array}$
$\begin{array}{c} \begin{array}{ccc} R_{3}^{3} & R_{4}^{3} & R_{1}^{3} \end{array}\\ \begin{array}{ccc} R_{3}^{3}=R_{4}^{3} & R_{4}^{3}=R_{1}^{3} & R_{3}^{3}=R_{1}^{3} \end{array}\\ R_{3}^{3}=R_{4}^{3}=R_{1}^{3} \end{array}$	$\begin{array}{c} \begin{array}{ccc} 1\;/\;2 & 1\;/\;2 & 1\;/\;2 \end{array}\\ \begin{array}{ccc} 1\;/\;4 & 1\;/\;4 & 1\;/\;4 \end{array}\\ 3\;/\;20 \end{array}$	$\begin{array}{c} \begin{array}{ccc} 1\;/\;2 & 1\;/\;2 & 1\;/\;2 \end{array}\\ \begin{array}{ccc} 1\;/\;4 & 1\;/\;4 & 1\;/\;4 \end{array}\\ 1\;/\;5 \end{array}$	$\begin{array}{c} \begin{array}{ccc} 1\;/\;2 & 1\;/\;2 & 1\;/\;2 \end{array}\\ \begin{array}{ccc} 1\;/\;4 & 1\;/\;4 & 1\;/\;4 \end{array}\\ 3\;/\;20 \end{array}$	$\begin{array}{c} \begin{array}{ccc} 1\;/\;2 & 1\;/\;2 & 1\;/\;2 \end{array}\\ \begin{array}{ccc} 1\;/\;4 & 1\;/\;4 & 1\;/\;4 \end{array}\\ 1\;/\;5 \end{array}$
$\begin{array}{c} \begin{array}{ccc} R_{4}^{4} & R_{1}^{4} & R_{2}^{4} \end{array}\\ \begin{array}{ccc} R_{4}^{4}=R_{1}^{4} & R_{1}^{4}=R_{2}^{4} & R_{4}^{4}=R_{2}^{4} \end{array}\\ R_{4}^{4}=R_{1}^{4}=R_{2}^{4} \end{array}$	$\begin{array}{c} \begin{array}{ccc} 1\;/\;2 & 1\;/\;2 & 1\;/\;2 \end{array}\\ \begin{array}{ccc} 1\;/\;4 & 1\;/\;4 & 1\;/\;4 \end{array}\\ 3\;/\;20 \end{array}$	$\begin{array}{c} \begin{array}{ccc} 1\;/\;2 & 1\;/\;2 & 1\;/\;2 \end{array}\\ \begin{array}{ccc} 1\;/\;4 & 1\;/\;4 & 1\;/\;4 \end{array}\\ 3\;/\;20 \end{array}$	$\begin{array}{c} \begin{array}{ccc} 1\;/\;2 & 1\;/\;2 & 1\;/\;2 \end{array}\\ \begin{array}{ccc} 1\;/\;4 & 1\;/\;4 & 1\;/\;4 \end{array}\\ 3\;/\;20 \end{array}$	$\begin{array}{c} \begin{array}{ccc} 1\;/\;2 & 1\;/\;2 & 1\;/\;2 \end{array}\\ \begin{array}{ccc} 1\;/\;4 & 1\;/\;4 & 1\;/\;4 \end{array}\\ 1\;/\;5 \end{array}$

**Table A6:** Distributions in the selection of the disturbed hypercyclic systems used. The format is the same as in Table A3.

	$H_{0}^{\prime }$	$H_{1}^{\prime }$	$H_{2}^{\prime }$	$H_{3}^{\prime }$	$H_{4}^{\prime }$
$\begin{array}{c} \begin{array}{ccc} R_{1}^{1} & R_{2}^{1} & R_{3}^{1} \end{array}\\ \begin{array}{ccc} R_{1}^{1}=R_{2}^{1} & R_{2}^{1}=R_{3}^{1} & R_{1}^{1}=R_{3}^{1} \end{array}\\ R_{1}^{1}=R_{1}^{1}=R_{3}^{1} \end{array}$	23231213131316	13122316131316	1414140000	35351525151515	35351525151515
$\begin{array}{c} \begin{array}{ccc} R_{2}^{2} & R_{3}^{2} & R_{4}^{2} \end{array}\\ \begin{array}{ccc} R_{2}^{2}=R_{3}^{2} & R_{3}^{2}=R_{4}^{2} & R_{2}^{2}=R_{4}^{2} \end{array}\\ R_{2}^{2}=R_{3}^{2}=R_{4}^{2} \end{array}$	12121316131316	23231213131316	12141414000	35252515152515	35253515152515
$\begin{array}{c} \begin{array}{ccc} R_{3}^{3} & R_{4}^{3} & R_{1}^{3} \end{array}\\ \begin{array}{ccc} R_{3}^{3}=R_{4}^{3} & R_{4}^{3}=R_{1}^{3} & R_{3}^{3}=R_{1}^{3} \end{array}\\ R_{3}^{3}=R_{4}^{3}=R_{1}^{3} \end{array}$	12121313131316	23122313131316	121412014140	252535015150	2535351515150
$\begin{array}{c} \begin{array}{ccc} R_{4}^{4} & R_{1}^{4} & R_{2}^{4} \end{array}\\ \begin{array}{ccc} R_{4}^{4}=R_{1}^{4} & R_{1}^{4}=R_{2}^{4} & R_{4}^{4}=R_{2}^{4} \end{array}\\ R_{4}^{4}=R_{1}^{4}=R_{2}^{4} \end{array}$	12121313131316	12232313131316	34141214141214	35253515152515	35353525152515

## Appendix B

Here, we present the distributions of the random variables in each context of the systems W and GHZ (Table A7). Because our claim that the W is noncontextual at level 2 seems to contradict the claim made in Ref. [12], we present in Table A8 the distribution of a reduced coupling of W from which one can compute the probabilities shown in Table A7. For instance, in Table A7,

$$P\left[A_{1}^{2}=B_{2}^{2}=1\right]=\frac{1}{6}.$$

By definition of the reduced coupling, we should have

$$P\left[A_{1}^{2}=B_{2}^{2}=1\right]=P\left[S_{1}=T_{2}=1\right].$$

From Table A8, we find

$$P\left[S_{1}=T_{2}=1\right]=P\left[\begin{array}{c} S_{1}\\ {\bar{S}}_{2}\\ {\bar{S}}_{3}\\ T_{1}\\ T_{2}\\ T_{3} \end{array}\right]+P\left[\begin{array}{c} S_{1}\\ {\bar{S}}_{2}\\ {\bar{S}}_{3}\\ {\bar{T}}_{1}\\ T_{2}\\ T_{3} \end{array}\right]=\frac{1}{8}+\frac{1}{24}=\frac{1}{6}.$$

**Table A7:** Distribution of variables in the W and GHZ systems. The format is the same as in Table A3.

	W	GHZ
$\begin{array}{c} \begin{array}{ccc} A_{1}^{1} & A_{2}^{1} & A_{3}^{1} \end{array}\\ \begin{array}{ccc} A_{1}^{1}=A_{2}^{1} & A_{2}^{1}=A_{3}^{1} & A_{1}^{1}=A_{3}^{1} \end{array}\\ A_{1}^{1}=A_{2}^{1}=A_{3}^{1} \end{array}$	1313130000	12121214141414
$\begin{array}{c} \begin{array}{ccc} A_{1}^{2} & B_{2}^{2} & B_{3}^{2} \end{array}\\ \begin{array}{ccc} A_{1}^{2}=B_{2}^{2} & B_{2}^{2}=B_{3}^{2} & A_{1}^{2}=B_{3}^{2} \end{array}\\ A_{1}^{2}=B_{2}^{2}=B_{3}^{2} \end{array}$	1312121651216112	1212121414140
$\begin{array}{c} \begin{array}{ccc} B_{1}^{3} & A_{2}^{3} & B_{3}^{3} \end{array}\\ \begin{array}{ccc} B_{1}^{3}=A_{2}^{3} & A_{2}^{3}=B_{3}^{3} & B_{1}^{3}=B_{3}^{3} \end{array}\\ B_{1}^{3}=A_{2}^{3}=B_{3}^{3} \end{array}$	1213121616512112	1212121414140
$\begin{array}{c} \begin{array}{ccc} B_{1}^{4} & B_{2}^{4} & A_{3}^{4} \end{array}\\ \begin{array}{ccc} B_{1}^{4}=B_{2}^{4} & B_{2}^{4}=A_{3}^{4} & B_{1}^{4}=A_{3}^{4} \end{array}\\ B_{1}^{4}=B_{2}^{4}=A_{3}^{4} \end{array}$	1212135121616112	1212121414140
$\begin{array}{c} \begin{array}{ccc} A_{1}^{5} & A_{2}^{5} & B_{3}^{5} \end{array}\\ \begin{array}{ccc} A_{1}^{5}=A_{2}^{5} & A_{2}^{5}=B_{3}^{5} & A_{1}^{5}=B_{3}^{5} \end{array}\\ A_{1}^{5}=A_{2}^{5}=B_{3}^{5} \end{array}$	131312016160	
$\begin{array}{c} \begin{array}{ccc} A_{1}^{6} & B_{2}^{6} & A_{3}^{6} \end{array}\\ \begin{array}{ccc} A_{1}^{6}=B_{2}^{6} & B_{2}^{6}=A_{3}^{6} & A_{1}^{6}=A_{3}^{6} \end{array}\\ A_{1}^{6}=B_{2}^{6}=A_{3}^{6} \end{array}$	131213161600	
$\begin{array}{c} \begin{array}{ccc} B_{1}^{7} & A_{2}^{7} & A_{3}^{7} \end{array}\\ \begin{array}{ccc} B_{1}^{7}=A_{2}^{7} & A_{2}^{7}=A_{3}^{7} & B_{1}^{7}=A_{3}^{7} \end{array}\\ B_{1}^{7}=A_{2}^{7}=A_{3}^{7} \end{array}$	121313160160	
$\begin{array}{c} \begin{array}{ccc} B_{1}^{8} & B_{2}^{8} & B_{3}^{8} \end{array}\\ \begin{array}{ccc} B_{1}^{8}=B_{2}^{8} & B_{2}^{8}=B_{3}^{8} & B_{1}^{8}=B_{3}^{8} \end{array}\\ B_{1}^{8}=B_{2}^{8}=B_{3}^{8} \end{array}$	12121251251251238	

**Table A8:** Distribution of a reduced coupling $\left(S_{1},S_{2},S_{3},T_{1},T_{2},T_{3}\right)$ of system W (with *S* and *T* being distributional copies of *A* and B, respectively). The events whose probabilities are shown in the bottom row are values of the reduced coupling $\left[S_{1}=\pm 1,S_{2}=\pm 1,S_{3}=\pm 1,T_{1}=\pm 1,T_{2}=\pm 1,T_{3}=\pm 1\right],$ shown in the following way: $S_{i}$ indicates $S_{i}=1$ and ${\bar{S}}_{i}$ indicates $S_{i}=-1$; analogously for $T_{j}$ and ${\bar{T}}_{j}$. All values of the reduced coupling not explicitly shown have probability zero.

**Value** **of** **Coupling**	$\begin{array}{c} S_{1}\\ {\bar{S}}_{2}\\ {\bar{S}}_{3}\\ T_{1}\\ T_{2}\\ T_{3} \end{array}$	$\begin{array}{c} S_{1}\\ {\bar{S}}_{2}\\ {\bar{S}}_{3}\\ {\bar{T}}_{1}\\ T_{2}\\ T_{3} \end{array}$	$\begin{array}{c} S_{1}\\ {\bar{S}}_{2}\\ {\bar{S}}_{3}\\ T_{1}\\ {\bar{T}}_{2}\\ {\bar{T}}_{3} \end{array}$	$\begin{array}{c} S_{1}\\ {\bar{S}}_{2}\\ {\bar{S}}_{3}\\ {\bar{T}}_{1}\\ {\bar{T}}_{2}\\ {\bar{T}}_{3} \end{array}$	$\begin{array}{c} {\bar{S}}_{1}\\ S_{2}\\ {\bar{S}}_{3}\\ T_{1}\\ T_{2}\\ T_{3} \end{array}$	$\begin{array}{c} {\bar{S}}_{1}\\ S_{2}\\ {\bar{S}}_{3}\\ {\bar{T}}_{1}\\ {\bar{T}}_{2}\\ {\bar{T}}_{3} \end{array}$	$\begin{array}{c} {\bar{S}}_{1}\\ {\bar{S}}_{2}\\ S_{3}\\ T_{1}\\ T_{2}\\ T_{3} \end{array}$	$\begin{array}{c} {\bar{S}}_{1}\\ {\bar{S}}_{2}\\ S_{3}\\ T_{1}\\ T_{2}\\ {\bar{T}}_{3} \end{array}$	$\begin{array}{c} {\bar{S}}_{1}\\ {\bar{S}}_{2}\\ S_{3}\\ {\bar{T}}_{1}\\ T_{2}\\ {\bar{T}}_{3} \end{array}$	$\begin{array}{c} {\bar{S}}_{1}\\ {\bar{S}}_{2}\\ S_{3}\\ T_{1}\\ {\bar{T}}_{2}\\ T_{3} \end{array}$	$\begin{array}{c} {\bar{S}}_{1}\\ {\bar{S}}_{2}\\ S_{3}\\ {\bar{T}}_{1}\\ {\bar{T}}_{2}\\ T_{3} \end{array}$	$\begin{array}{c} {\bar{S}}_{1}\\ {\bar{S}}_{2}\\ S_{3}\\ {\bar{T}}_{1}\\ {\bar{T}}_{2}\\ {\bar{T}}_{3} \end{array}$	*…*
P	18	124	124	18	16	16	112	124	124	124	124	112	0
